# Supplementary material for: Evaluation of Lower Leg Arteries and Fibular Perforators before Microsurgical Fibular Transfer Using Noncontrast-Enhanced Quiescent-Interval Slice-Selective (QISS) Magnetic Resonance Angiography
Source: J Clin Med. 2023 Feb 18;12(4):1634. doi: 10.3390/jcm12041634 (PMC9964888; doi:10.3390/jcm12041634)
Supplement: Supplementary file 1 [file jcm-12-01634-s001.zip › jcm-2136314-supplementary.pdf]

Supplementary Files

Table S1. Protocol parameters of noncontrast enhanced 2D ECG-triggered QISS-MRA.

Parameters	Values/Setting
**Imaging mode**	2D
**TR (ms)**	667.6
**TE (ms)**	1.7
**Acquisition matrix (Px)**	400 × 400
**Reconstructed pixel (mm^2)**	0.5 × 0.5
**In-plane interpolation**	On
**Slice thickness (mm)**	3
**Number of slices per slab**	60
**Slice distance factor (%)**	-20
**Number of averages**	1
**Receiver bandwidth (Hz/Px)**	658
**Flip angle (°)**	120
**Slice orientation**	Transverse
**Phase oversampling (%)**	0
**Filter**	Raw data, distortion correction (2D); prescan normalizer
**B_0 shim mode**	Standard

**Asymmetrical echo**	Weak
**RF pulse type**	Normal
**pulse length (ms)**	2.56
**Gradient mode**	Fast
**Maximum amplitude (mT/m)**	24
**Maximum rise time (µs/mT/s)**	5.55
**Maximum slew rate (mT/m/ms)**	180.2
**iPAT modus (acceleration factor/number of reference lines)**	GRAPPA (2/24)
**Partial Fourier (phase and slice)**	5/8
**Thickness of venous saturation slab (mm)**	75
**TI (ms)**	345
**TD (ms)**	100
**Acquisition time per slice (s)**	<1<br>Depended on the cardiac cycle

TR = repetition time, TE = echo time, Px = pixel, Hz = Hertz, RF = radiofrequency, iPAT = integrated parallel imaging technique, GRAPPA = GeneRalized Autocalibrating Partial Parallel Acquisition, TI = time from in-plane and venous saturation to acquisition of central k-space (ky=0), TD = trigger delay.

Calculation of the apparent signal-to-noise ratio (SNR) and the apparent contrast-to-noise ratio (CNR)

Nine circular regions of interest (ROIs) of appropriate size in relation to the cross-sectional areas of the corresponding vessels were placed in the aorta (ROI_{Aorta}, 2.0 cm above the aortic bifurcation with a size of 100 mm^2), in the right and left popliteal artery (ROI_{PA}, at the level of the knee joint space, 20 mm^2), and in the three arteries of the right and left lower leg (ROI_{AT}, ROI_{PT}, ROI_{FA}, each at the same level, measured 1 cm below the bifurcation of the tibiofibular trunk, 6 mm^2). Here, the anterior tibial artery is abbreviated as AT, posterior tibial artery as PT, and fibular artery as FA.

In addition, three ROI_{Air} were drawn in the background without visual imaging artifacts at the same level of ROI_{Aorta}, ROI_{PA} and $ROI_{AT/PT/FA}$ to determine the SNR. All three ROI_{Air} were the same size as the ROI in the corresponding artery. In addition, two ROIs were placed in the right and left tibialis anterior muscle (ROI_{Muscle}, 6 mm^2) to calculate the CNR of AT, PT, and FA.

Table S2. Correlation of different variables with surgical complications.

Variable	Phi-Value	Rho-Value	*p*-Value
Gender	0.007		1
Hypercholesterolemia	-0.021		1
Smoking	0.007		1
Arterial hypertension	0.021		1
Diabetes	-0.021		1
Obesity	-0.226		0.36
Grading of Right FA		-0.026	0.86
Grading of Left FA		-0.014	0.93
Presence of Perforators Right Leg		0.162	0.27
Presence of Perforators Left Leg		0.226	0.11
Number of Perforators Right Leg		0.103	0.49
Number of Perforators Left Leg		0.184	0.20
